# Health Galaxy: A Multi-organ Trajectory-Computing Framework in Type 2 Diabetes

**DOI:** 10.34133/research.1378

**Published:** 2026-07-23

**Authors:** Han Lv, Jia Li, Dong Li, Bin Sheng, Zhenchang Wang

**Affiliations:** ^1^ Department of Radiology, Beijing Friendship Hospital, Capital Medical University, Beijing 100050, China.; ^2^ Department of Health Data Science, School of Medical Technology, Capital Medical University, Beijing 100050, China.; ^3^ Medical Digital Intelligence Innovation Center, Beijing Friendship Hospital, Capital Medical University, Beijing 100050, China.; ^4^ Medical Data Science Center, Tsinghua University Affiliated Beijing Tsinghua Changgung Hospital, Beijing 102218, China.; ^5^ School of Computer Science, Shanghai Jiao Tong University, Shanghai 200240, China.

## Abstract

Health research is shifting from isolated risk snapshots toward longitudinal, multimodal representations of disease. In type 2 diabetes, patients with comparable glycemic control may follow sharply divergent renal, cardiovascular, hepatic, and neurocognitive courses—heterogeneity that single-endpoint, single-window risk models cannot resolve. Here, we propose the Health Galaxy, a state-space framework that represents each individual by multi-organ position and direction of motion over time, integrating organ-aging clocks, cross-organ coupling, and trajectory-aware generative modeling. We operationalize this framework through 3 falsifiable structural units—clusters, channels, and hubs—that can be tested for reproducibility across cohorts and modalities. The framework reframes intervention as trajectory rerouting rather than marker reduction, with type 2 diabetes as a tractable first test case.

## Why a State-Space View, and Why Now

In the clinic, 3 patients with type 2 diabetes (T2D) and identical hemoglobin A1c (HbA1c) values may both be told that their disease is “well controlled”, yet years later, one progresses to kidney failure, another develops heart failure, and a third drifts toward steatotic liver disease, retinopathy, neuropathy, or cognitive decline. Glycemic targets, estimated glomerular filtration rate (eGFR), albuminuria, lipids, blood pressure, and cardiovascular risk scores are organized as a network spanning multiple organs and successive time points, giving rise to a spatial trajectory of the disease in a high-dimensional state space; how to harness these high-dimensional features, however, remains to be explored [1–3].

Advances in organ-aging clocks, electronic health records, imaging, continuous glucose monitoring, wearables, and medication histories now make it possible to observe the diabetic body repeatedly across time and across organ systems. Combined with artificial intelligence (AI)-based analytics, these heterogeneous, time-stamped data support the dynamic representation of multi-organ disease in motion, going beyond the cross-sectional snapshots that conventional markers provide [1,4–6]. A new framework is therefore needed to organize them into a single, interpretable representation of where each organ system is heading and how fast.

**Fig. 1.**
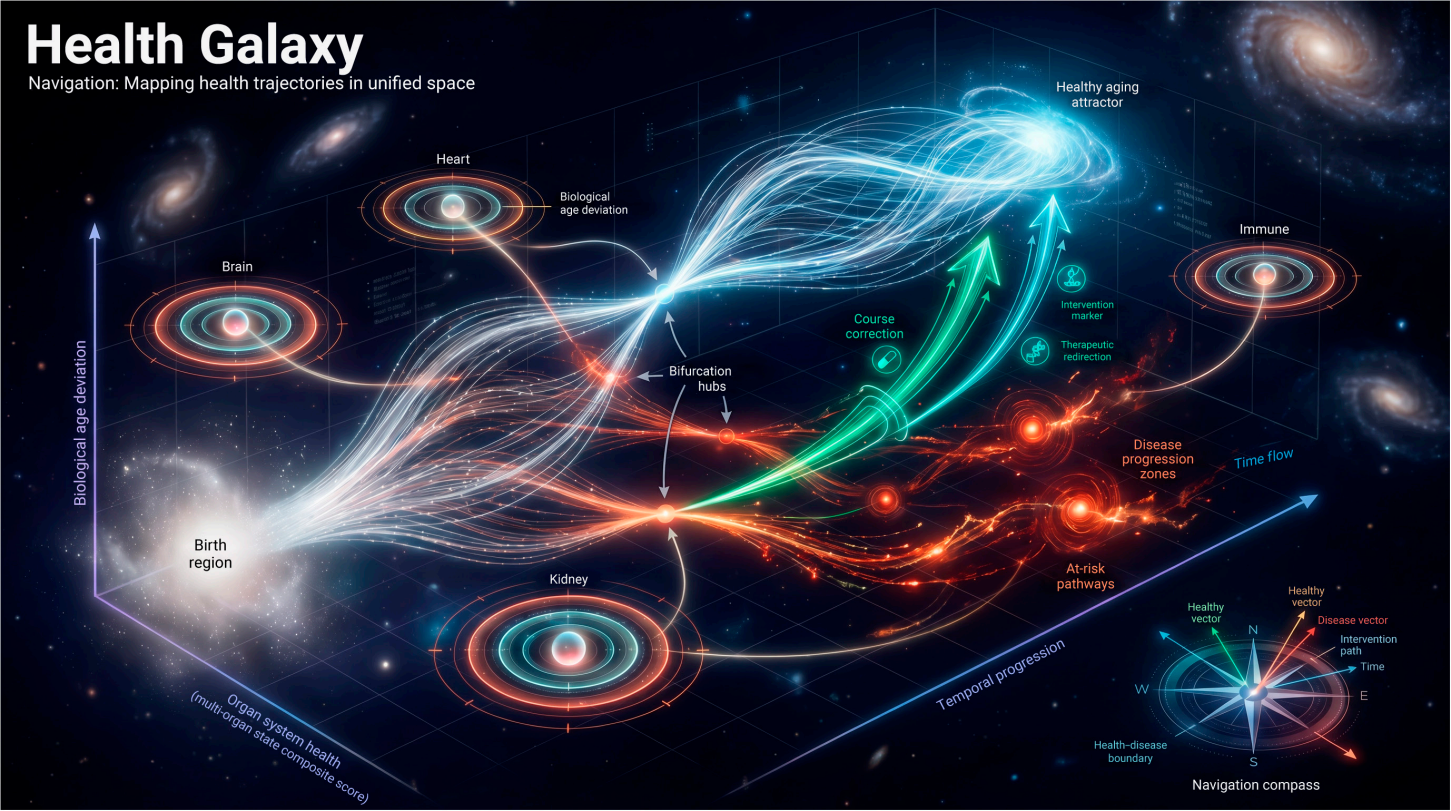
Conceptual architecture of the Health Galaxy representing individual multi-organ trajectories in a shared state space to support trajectory-based risk stratification and intervention-driven course correction in type 2 diabetes.

Therefore, we introduce the concept of the Health Galaxy as a framework that conceptualizes T2D as a trajectory through a shared, high-dimensional multi-organ state space—thereby complementing molecular omics, which has transformed our understanding of diabetic pathophysiology yet do not by itself captures these longitudinal, organ-level dynamics (Fig. 1) [2,3]. Accordingly, we propose the Health Galaxy as a state-space framework that represents each individual, at each visit, as a computable multi-organ state *X*(*t*), assigning each person a position in a shared landscape and following the direction and speed of their movement rather than reducing them to a single future probability.

## Biological Foundations: Asynchronous Organ Aging and Interorgan Cross Talk

Basic biology has already provided the key insight, and it has proven feasible at the molecular level. Two bodies of evidence give the Health Galaxy its grounding. The first is asynchronous organ aging: organ-aging clock studies have shown that plasma proteomic, imaging, or multi-omic signatures are associated with chronic disease and mortality, so that a person whose kidney or heart appears biologically older than expected faces elevated organ-specific risk [1,4,6]. The second is interorgan cross talk: in T2D, adipose tissue, the vasculature, the heart, the retina, the brain, and the immune system communicate through metabolites, hormones, inflammatory mediators, and neural signals, so that disease in one system accelerates decline in others [7]. Recent work unifies these ideas, reframing isolated aging clocks as a coupled organ network in which age-driven asynchrony and interorgan interaction jointly shape mortality risk [8]. These insights, however, were established at the molecular scale. The pressing question is how to carry them into routine phenotypic and physiological data—the measurements thar clinics actually collect—and turn them into a macro-level, computable representation.

## Analytical Foundations of the Health Galaxy

The framework continuously assembles multimodal, time-stamped data—glycemic and metabolic laboratory measures; renal and hepatic panels; blood pressure; lipids; imaging of the heart, liver, kidney, retina, and brain; omics-derived organ clocks; continuous glucose monitoring; wearables; and medication and adherence records—and aligns them through time normalization, event-based alignment, interoperable standards, and standardized preprocessing so that heterogeneous, irregularly sampled inputs become comparable states. From the resulting trajectories, machine learning, network science, and causal-inference methods support 3 complementary goals: trajectory-based risk stratification (forecasting the proximity and ordering of competing complications), mechanism-informed inference (estimating organ-coupling structure and risk transmission), and intervention optimization (simulating counterfactual or treatment-conditioned trajectories). Trajectory-aware generative models may help represent plausible future paths, but such simulations are clinically meaningful only when they are calibrated, uncertainty aware, externally validated, and transportable across populations and care settings [9,10].

## Applications of the Health Galaxy

Example 1: Distinguishing divergent futures under similar control. Consider 2 patients with comparable HbA1c, eGFR, and baseline cardiovascular risk. In the Health Galaxy, their states differ along axes that conventional scores collapse—rate of albuminuria rise, trajectory of cardiac and vascular clocks, hepatic fat dynamics, and the coupling between renal and cardiac decline. One patient’s trajectory accelerates along a renal–cardiac channel toward kidney failure and heart failure, while the other’s drifts toward a hepatic–metabolic path. By making these divergent trajectories explicit and quantifying organ coupling, the framework supports earlier, organ-specific surveillance and individualized follow-up intervals that a single shared HbA1c target cannot provide [2,7,8].

Example 2: From forecasting to redirection. The framework’s distinctive aim is to model whether a trajectory can be changed. For a patient whose state is moving rapidly along a renal–cardiac channel, the Health Galaxy can be used to simulate the expected effect of initiating a sodium–glucose cotransporter-2 inhibitor or glucagon-like peptide-1 receptor agonist, intensifying blood-pressure control, or weight reduction and to monitor—after the intervention—whether the realized trajectory velocity actually slows. This shifts the clinical question from “what is this patient’s risk?” to “did this action bend the path, and by how much?”

## Critical Challenges and Future Directions

Several challenges must be resolved before the Health Galaxy can be deployed. First, multimodal diabetes data are unevenly sampled and shaped by access, imaging availability, device adherence, prescribing patterns, and follow-up frequency, so apparent trajectories may reflect workflow rather than biology; standardized acquisition, missing-data modeling, and modality ablation are essential. Second, organ clocks may encode disease burden or treatment exposure, demanding cautious, theory-informed interpretation rather than literal reading of organ age. Third, trajectory and generative models remain limited in transparency and generalizability and are vulnerable to population bias and distribution shift as assays, imaging protocols, populations, and diabetes therapies evolve, making explainability, uncertainty quantification, external validation, drift monitoring, and prospective testing design requirements rather than afterthoughts [10]. Finally, continuous, context-rich monitoring raises governance questions of data ownership, individual agency, and fairness that must be addressed under recognized standards.
